# Melanocortin peptides inhibit urate crystal-induced activation of phagocytic cells

**DOI:** 10.1186/ar2827

**Published:** 2009-10-08

**Authors:** Franco Capsoni, Anna Maria Ongari, Eva Reali, Anna Catania

**Affiliations:** 1 Rheumatology Unit, Istituto Ortopedico Galeazzi IRCCS (Istituto Di Ricovero e Cura a Carattere Scientifico), University of Milan, Via Riccardo Galeazzi 4, 20161 Milan, Italy; 2 INGM-National Institute of Molecular Genetics, Fondazione IRCCS Ospedale Maggiore Policlinico, Mangiagalli e Regina Elena, Via Francesco Sforza 28, 20122 Milan, Italy; 3 Center for Preclinical Investigation, Fondazione IRCCS Ospedale Maggiore Policlinico, Mangiagalli e Regina Elena, Via Francesco Sforza 28, 20122 Milan, Italy

## Abstract

**Introduction:**

The melanocortin peptides have marked anti-inflammatory potential, primarily through inhibition of proinflammatory cytokine production and action on phagocytic cell functions. Gout is an acute form of arthritis caused by the deposition of urate crystals, in which phagocytic cells and cytokines play a major pathogenic role. We examined whether alpha-melanocyte-stimulating hormone (α-MSH) and its synthetic derivative (CKPV)_2 _influence urate crystal-induced monocyte (Mo) activation and neutrophil responses *in vitro*.

**Methods:**

Purified Mos were stimulated with monosodium urate (MSU) crystals in the presence or absence of melanocortin peptides. The supernatants were tested for their ability to induce neutrophil activation in terms of chemotaxis, production of reactive oxygen intermediates (ROIs), and membrane expression of CD11b, Toll-like receptor-2 (TLR2) and TLR4. The proinflammatory cytokines interleukin (IL)-1β, IL-8, and tumor necrosis factor-alpha (TNF-α) and caspase-1 were determined in the cell-free supernatants. In parallel experiments, purified neutrophils were preincubated overnight with or without melanocortin peptides before the functional assays.

**Results:**

The supernatants from MSU crystal-stimulated Mos exerted chemoattractant and priming activity on neutrophils, estimated as ROI production and CD11b membrane expression. The supernatants of Mos stimulated with MSU in the presence of melanocortin peptides had less chemoattractant activity for neutrophils and less ability to prime neutrophils for CD11b membrane expression and oxidative burst. MSU crystal-stimulated Mos produced significant levels of IL-1β, IL-8, TNF-α, and caspase-1. The concentrations of proinflammatory cytokines, but not of caspase-1, were reduced in the supernatants from Mos stimulated by MSU crystals in the presence of melanocortin peptides. Overnight incubation of neutrophils with the peptides significantly inhibited their ability to migrate toward chemotactic supernatants and their capacity to be primed in terms of ROI production.

**Conclusions:**

α-MSH and (CKPV)_2 _have a dual effect on MSU crystal-induced inflammation, inhibiting the Mos' ability to produce neutrophil chemoattractants and activating compounds and preventing the neutrophil responses to these proinflammatory substances. These findings reinforce previous observations on the potential role of α-MSH and related peptides as a new class of drugs for treatment of inflammatory arthritis.

## Introduction

Alpha-melanocyte-stimulating hormone (α-MSH) is an endogenous tridecapeptide with multiple effects on host cells. The synthetic peptide inhibits inflammatory responses in experimental models of acute and chronic disorders, including bowel diseases, allergy, adjuvant arthritis, and sepsis [1-4]. α-MSH interacts with host cells through recognition of specific melanocortin receptors (MCRs 1 to 5). Its anti-inflammatory action depends primarily on inhibition of cytokine production by target cells. This is achieved by preventing the activation of nuclear transcription factor-kappa-B (NF-κB) (reviewed in [4]). Several leukocyte functions, including reactive oxygen intermediate (ROI) generation and release of proteolytic enzymes, are also influenced by α-MSH. Nitric oxide production and the expression of adhesion molecules are likewise inhibited in both neutrophils and monocytes (Mos) [5,6]. α-MSH inhibits human neutrophil migration and several other interleukin-8 (IL-8)-induced biological responses [7-9]; inhibition of antigen-stimulated lymphocyte proliferation has been reported also [10]. The significant role of α-MSH and related peptides in immune/inflammatory responses and their ability to prevent inflammation-mediated tissue injury suggest these molecules as a potential new class of anti-inflammatory drugs. However, with a view to this use, cost-effective stable analogs need to be developed.

Previous observations indicated that the anti-inflammatory message sequence of α-MSH [1-13] resides in the C-terminal tripeptide Lys-Pro-Val (MSH 11-13 or KPV) [11]. A dimer obtained by inserting a Cys-Cys linker between two units of KPV, (CKPV)_2_, inhibited tumor necrosis factor-alpha (TNF-α) production by lipopolysaccharide (LPS)-stimulated human leukocytes with potency similar to the stable α-MSH analog [Nle4-dPhe7]-α-MSH (NDP-α-MSH) and effectiveness greater than KPV. Effectiveness was similar *in vivo*: (CKPV)_2 _markedly inhibited circulating TNF-α after intravenous injection of LPS and significantly reduced TNF-α and NO_2_^- ^concentrations in plasma and in the peritoneal cavity in a rat model of LPS-induced peritonitis [12].

We recently reported that (CKPV)_2 _*in vitro *reduced several human neutrophil activities important for the host inflammatory response. These inhibitory effects included cell migration, ROI production, proinflammatory cytokine synthesis and secretion, and expression of adhesion molecules [9].

Gout is an acute form of arthritis causing substantial inflammation and involving tissue deposition of monosodium urate (MSU) crystals. Both Mos and neutrophils are important in pathogenesis. Recent observations suggest that MSU crystals act as danger signals with the ability to activate caspase-1 in an NALP-3 inflammosome-dependent manner, with production and release of active IL-1β [13]. In this model, the second phase of the inflammatory response is driven by the IL-1β/IL-1R pathway with activation of an MyD88-dependent signaling pathway and NF-κB activation followed by production of inflammatory mediators that elicit neutrophil recruitment into the joints, characteristic of the acute gouty inflammation [14].

The importance of Mos and neutrophils in the pathogenesis of acute gout suggests that α-MSH and related peptides control MSU-induced inflammation. This was suggested by Getting and colleagues [15,16], who showed that melanocortins had protective action in a rat model of gouty arthritis. Therefore, we examined whether α-MSH and the synthetic melanocortin (CKPV)_2 _influenced MSU crystal-induced human Mo activation and human neutrophil responses *in vitro*.

## Materials and methods

### Reagents

The peptides (CKPV)_2 _and α-MSH [1-13], N-acetylated and C-amidated, were kindly provided by Paolo Grieco, Department of Pharmaceutical and Toxicological Chemistry, University of Naples Federico II, Italy. Lymphoprep gradient (density 1.077 g/mL) and Nycoprep 1.068 gradient were purchased from Axis-Shield (Oslo, Norway). Extra-low endotoxin fetal bovine serum (FBS) was obtained from HyClone (Logan, UT, USA). Hanks' balanced salt solution, RPMI 1640, penicillin, streptomycin, glutamine, bovine serum albumin (BSA), *N*-formyl-methionyl-leucyl-phenylalanine (FMLP), luminol (5-amino-2,3-dihydro-1,4-phthalazinedione), and polymyxin B sulphate were from Sigma-Aldrich (St. Louis, MO, USA). Micropore filters were from Millipore Corporation (Bedford, MA, USA). The caspase-1 inhibitor z-YVAD-fmk was from Alexis Biochemicals (Farmingdale, NY, USA). The phycoerythrinated anti-Toll-like receptor-2 (TLR2), anti-TLR4, and anti-CD11b monoclonal antibodies (mAbs) were from eBioscence, Inc. (San Diego, CA, USA). Irrelevant class-matched mAbs used as controls for nonspecific binding were from Becton, Dickinson and Company (Franklin Lakes, NJ, USA). The mAbs anti-IL-1β and anti-IL-8 and the specific enzyme-linked immunosorbent assay (ELISA) for IL-1β, IL-8, TNF-α, and caspase-1 were from R&D Systems (Abingdon, UK). MSU crystals were prepared according to the method described by Murakami and colleagues [17]. The crystals were sterilized by heating at 180°C for 2 hours, were resuspended in phosphate-buffered saline (PBS) at a concentration of 10 mg/mL, and were verified free of endotoxin by the Limulus amoebocyte cell lysate assay (Sigma-Aldrich).

### Neutrophils and monocyte isolation and culture

Peripheral blood neutrophils were obtained by density gradient centrifugation (Lymphoprep) [18]. The purified cell population consisted of greater than 95% pure, viable neutrophils, assessed on the basis of morphology and Trypan blue exclusion. In some experiments, purified neutrophils, at the appropriate density (2.5 × 10^6^/mL), were incubated in polypropilene tissue culture tubes (Corning Incorporated, Corning, NY, USA) at 37°C in a humidified atmosphere of 5% CO_2 _for 18 hours in complete medium alone (see below), α-MSH 10^-6 ^M, or (CKPV)_2 _10^-6 ^M. At the end of incubation, neutrophils were used in the functional assays as described below. Viability of cells was always more than 95%.

Peripheral blood Mos were purified on a Nycoprep 1.068 gradient (as described in detail by Boyum [19]), which routinely yielded 85% to 90% Mo as assessed by Wright staining, non-specific esterase staining, and (in some cases) immunofluorescent staining for CD14; viability was more than 95% by Trypan blue exclusion. Freshly isolated Mos were resuspended in RPMI 1640 containing 10% heat-inactivated FBS (30 minutes at 56°C), 100 U/mL penicillin, 100 μg/mL streptomycin, and 2 mM glutamine (complete medium) at 1 × 10^6 ^Mos/mL. The Mo suspensions were incubated in polystyrene tissue culture flasks (cell growth area 25 cm^2^; Corning Incorporated) at 37°C in a 95% air-5% CO_2 _humidified atmosphere for 24 hours in complete medium with or without MSU crystals (1 mg/mL) and with or without the indicated concentrations of α-MSH or (CKPV)_2_.

To check for artifacts of trace endotoxin contamination of MSU crystals, the cells were incubated with MSU in the presence of polymyxin B sulphate (10 μg/mL) in some experiments. In three separate experiments, the caspase-1 inhibitor z-YVAD-fmk was added to the cultured cells at a final concentration of 10 μM. The supernatants of cultured Mos were collected by centrifugation (1,200 *g *for 15 minutes), were filtered through a 0.22-μm filter (Millipore Corporation), and were stored at -80°C until tested for their capacity to activate neutrophils and for their content of IL-1β, IL-8, TNF-α, and caspase-1. The concentration of MSU crystals we used (1 mg/mL) had been found to be optimal beforehand on a limited dose-response curve (0.1, 1.0, and 10.0 mg/mL; data not shown) and was similar to that used by others in similar experimental conditions [16,20,21]. The viability of cultured Mos, determined by Trypan blue exclusion, always exceeded 90%.

### Chemotaxis

Neutrophil chemotaxis was examined using a modified Boyden chamber assay with blind-well chambers and 3-μm micropore filters [22]. Briefly, 200 μL of the cell suspension containing 3.75 × 10^6 ^neutrophils/mL in RPMI 1640 + 0.4% BSA was layered on top of the filter, and the lower compartment was filled with 200 μL of the supernatants from MSU-stimulated Mos (SMMs) or the chemotactic factor FMLP (10^-8 ^M final concentration). After 120 minutes of incubation at 37°C in a humidified atmosphere with 5% CO_2_, the filters were fixed with ethanol and stained with hematoxylin-eosin. The chemotactic response was determined by counting the number of cells per high-power field which had migrated through the entire thickness of the filter; duplicate chambers were used in each experiment, and five fields were examined in each filter. In all cases, the person scoring the assay was blind to the experimental groupings. In some experiments, anti-IL-1β (1 μg/mL final concentration) or anti-IL-8 (4 μg/mL final concentration) or the two mAbs together were added to the SMMs before as chemoattractants.

### Chemiluminescence

Luminol-amplified chemiluminescence (CL) was used to examine the phagocyte production of ROIs in response to MSU crystals. To measure CL, 2.5 × 10^5 ^neutrophils were mixed in 3-mL polystyrene vials with 5 × 10^-5 ^M luminol in a final volume of 700 μL. The vials were placed in a Luminometer 1251 (LKB Wallac, Turku, Finland) and allowed to equilibrate in the dark for 5 minutes at 37°C, with intermittent shaking, and then the background light output was recorded in millivolts. MSU crystals (3 mg/mL final concentration) [23] were added using an appropriate dispenser (1291; LKB Wallac), and the CL was recorded continuously. The background counts were subtracted from the values obtained after neutrophil stimulation. To check the effect of the SMMs on CL production, neutrophils were preincubated with undiluted supernatants for 30 minutes at 37°C before the CL assay.

### Surface expression of CD11b, TLR2, and TLR4

Flow cytometry of purified neutrophils was used to determine the membrane expression of CD11b, TLR2, and TLR4. Phycoerythrin-conjugated mAbs were added to 100 μL of a purified cell suspension (2 × 10^5 ^cells in PBS containing 0.1% NaN_3 _and 10% human AB serum). The staining reactions were developed at 4°C for 30 minutes. After washing, the cells were analyzed by flow cytometry (Becton Dickinson FACS II; Becton, Dickinson and Company). A relative measure of antigen expression was obtained using the mean fluorescence intensity, converted from a log to a linear scale, after subtracting the cells' self-fluorescence and the fluorescence of cells incubated with irrelevant isotype control mAbs.

### Production of cytokines *in vitro*

IL-1β, IL-8, TNF-α, and caspase-1 protein concentrations were determined in the cell-free supernatants using specific ELISA in accordance with the procedures indicated by the manufacturer (R&D Systems).

### Statistical analysis

The data are expressed as mean ± standard error of the mean. Statistical analysis was done using the Student *t *test for unpaired or paired data, as appropriate. A probability of less than 0.05 was considered significant.

## Results

### Monosodium urate-stimulated monocytes produce neutrophil-activating mediators

**Figure 1:**
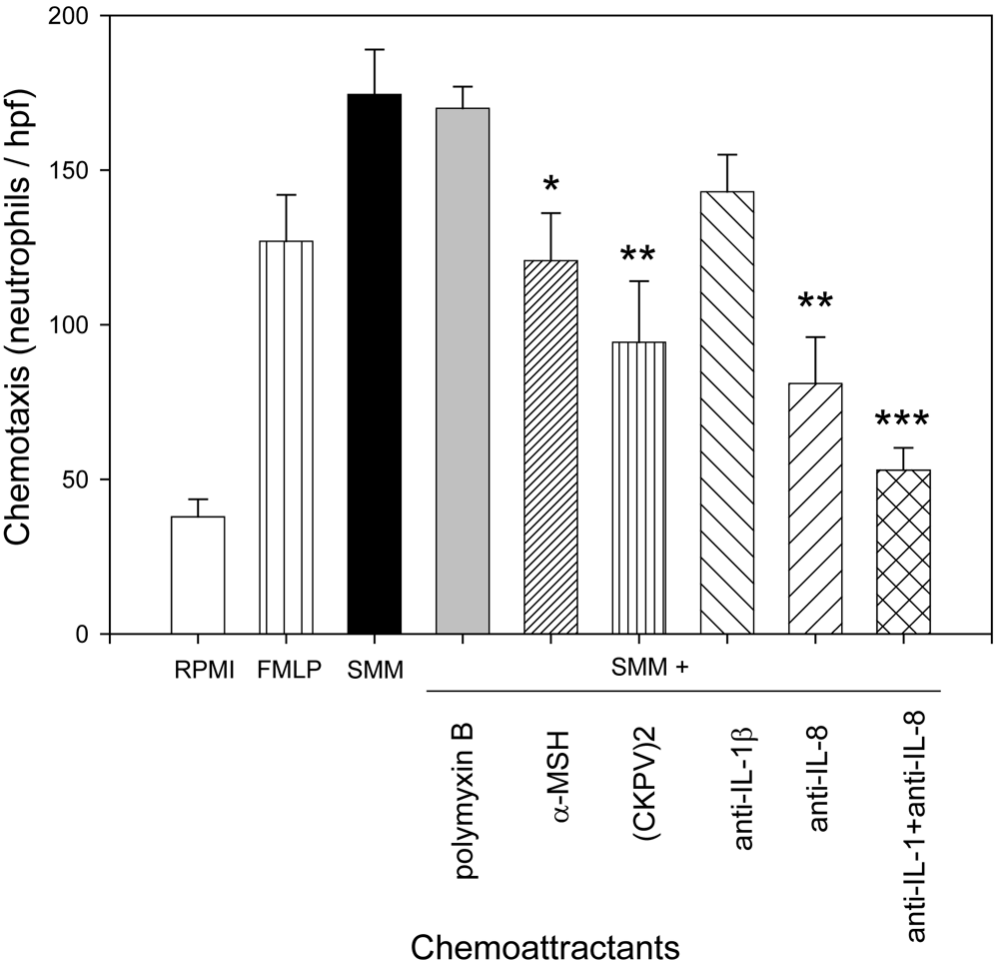
Chemotactic properties of supernatants from monosodium urate (MSU) crystal-stimulated monocytes (SMMs).  Monocytes were incubated for 18 hours with MSU crystals (1 mg/mL) with or without alpha-melanocyte-stimulating hormone (α-MSH) (10^-6 ^M) or (CKPV)_2 _(10^-6 ^M) or polymyxin B (10 μg/mL). The cell-free supernatants from 10 separate experiments (two with polymyxin B) were used as chemoattractants for purified human neutrophils. In three separate experiments, SMMs were preincubated (15 minutes at room temperature) with anti-interleukin-1 (anti-IL-1) or anti-IL-8 monoclonal antibodies or both before use as chemoattractants. The chemotactic activity of the standard chemoattractant *N*-formyl-methionyl-leucyl-phenylalanine (FMLP) (10^-8 ^M) is shown. Results are expressed as number of neutrophils per high-power field (hpf). Bars denote mean ± standard error of the mean. *P < 0.05; **P < 0.01; ***P < 0.005 versus SMMs.

**Figure 2:**
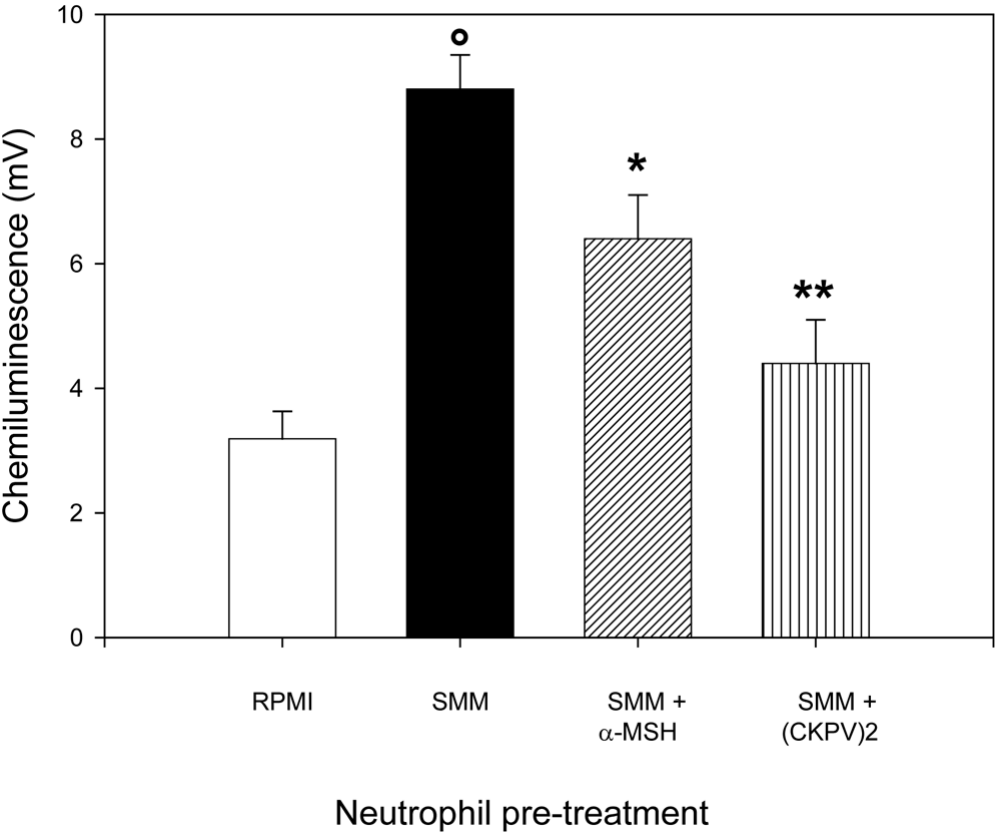
Priming activity of supernatants from monosodium urate (MSU) crystal-stimulated monocytes (SMMs) on chemiluminescence (CL) production by neutrophils.  Monocytes were incubated for 18 hours with MSU crystals (1 mg/mL) with or without alpha-melanocyte-stimulating hormone (α-MSH) (10^-6 ^M) or (CKPV)_2 _(10^-6 ^M). The cell-free supernatants were used to pretreat purified neutrophils (30 minutes at 37°C) before analysis of their CL production in response to MSU (3 mg/mL). Results are expressed as peak CL values in millivolts. Bars denote mean ± standard error of the mean. °*P *< 0.001 versus RPMI; *P < 0.05 and **P < 0.01 versus SMMs.

**Figure 3:**
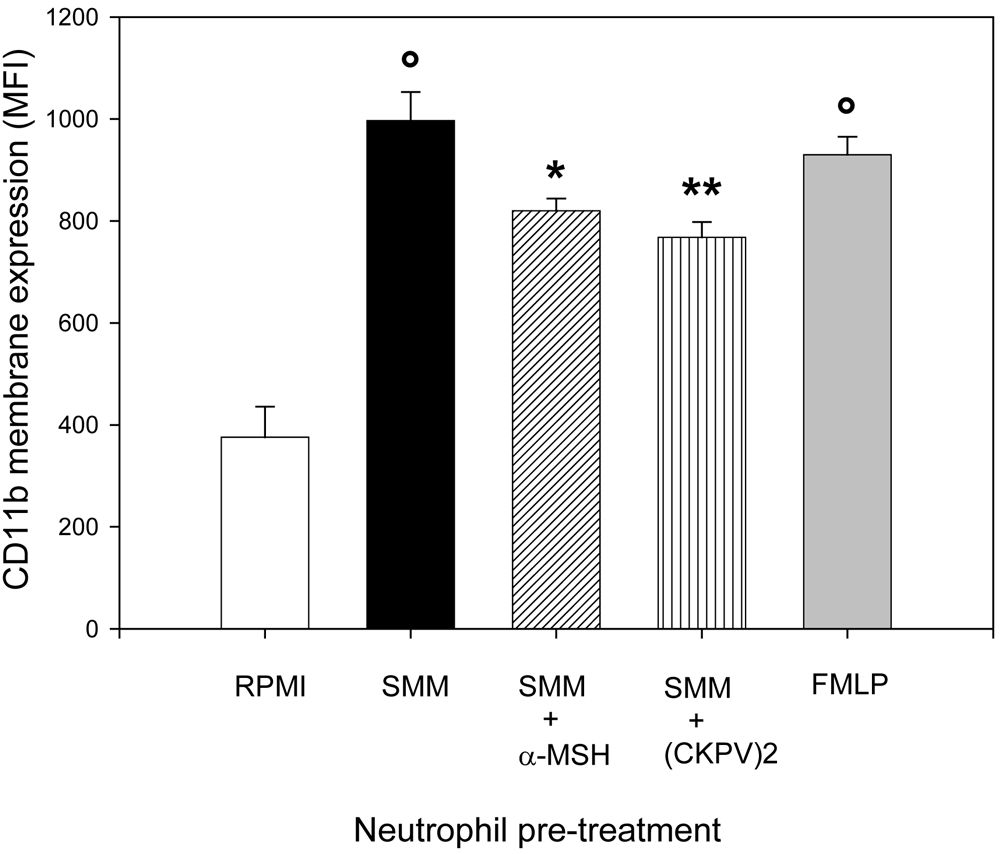
Activity of supernatants from monosodium urate (MSU) crystal-stimulated monocytes (SMMs) on CD11b membrane expression on human neutrophils.  Monocytes were incubated for 18 hours with MSU crystals (1 mg/mL) with or without alpha-melanocyte-stimulating hormone (α-MSH) (10^-6 ^M) or (CKPV)_2 _(10^-6 ^M). Purified neutrophils were pretreated with the different SMMs or *N*-formyl-methionyl-leucyl-phenylalanine (FMLP) (30 minutes at 37°C) before staining with the phycoerythrin-conjugated anti-CD11b monoclonal antibody. The results are expressed as mean fluorescence intensity (MFI) ± standard error of the mean, corrected for non-specific staining, from five separate experiments. °*P *< 0.001 versus RPMI; *P < 0.05 and **P < 0.01 versus SMMs.

Mos were incubated with MSU for 24 hours with or without α-MSH or (CKPV)_2_; the cell-free supernatants were then tested for their ability to induce neutrophil responses. The SMMs showed significant chemotactic activity for neutrophils; this was not due to contaminating LPS since the activity was no different in SMMs from Mos cultured with polymyxin B sulphate (Figure 1). The chemotactic activity of SMMs was similar to or even greater than the standard chemoattractant FMLP (Figure 1). MSU crystals induced CL production by neutrophils. These SMMs also had a priming effect on neutrophils: when neutrophils were preincubated with the SMMs, they showed an enhanced respiratory burst in response to a challenge with MSU (Figure 2). In addition, neutrophils pretreated with the SMMs showed an increase in the membrane expression of CD11b (Figure 3), whereas there was no change in TLR2 and TLR4 membrane expression (data not shown).

**Figure 4:**
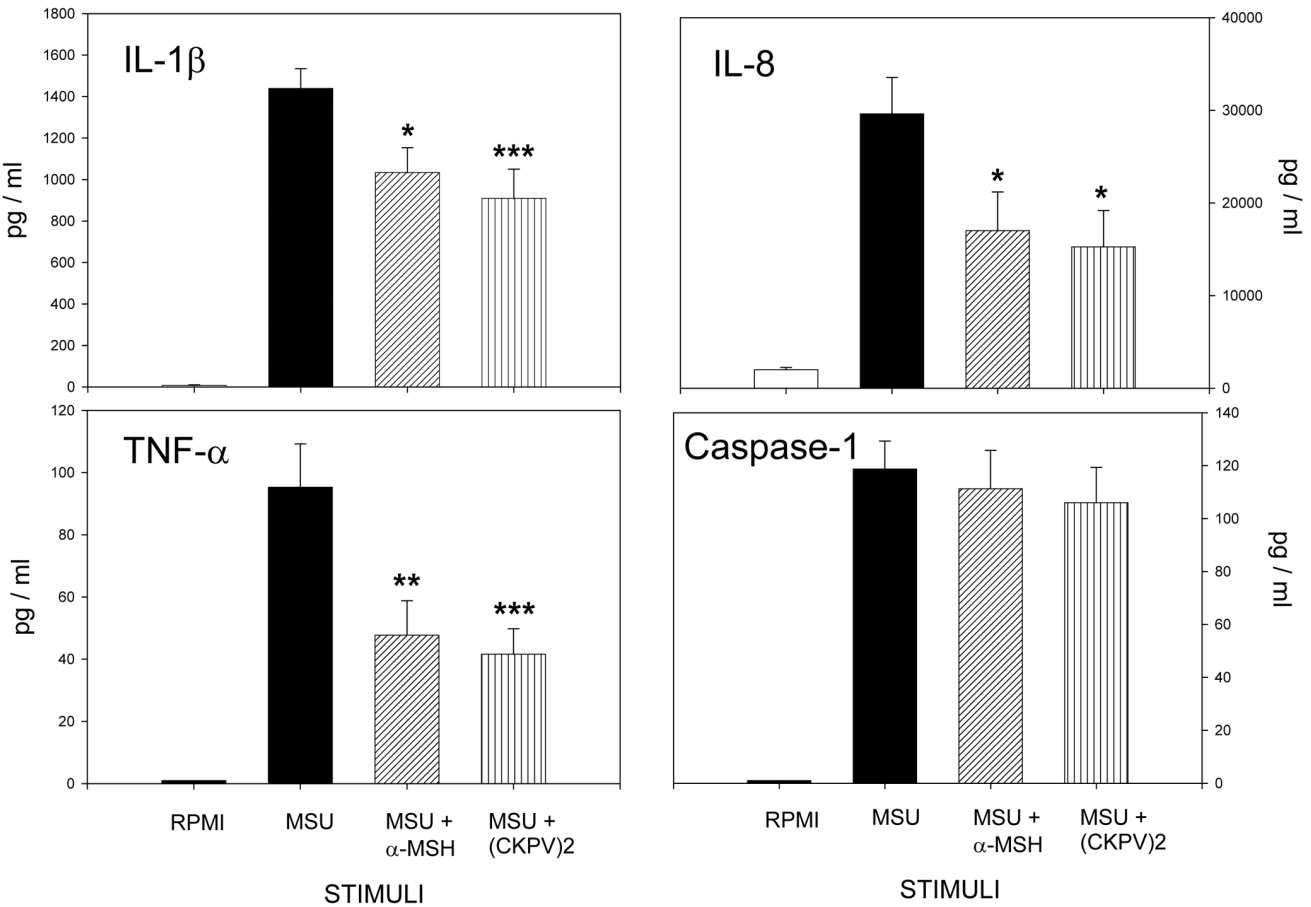
Effects of alpha-melanocyte-stimulating hormone (α-MSH) and (CKPV)_2 _on inflammatory mediator production by monosodium urate (MSU)-stimulated human monocytes.  Monocytes were incubated for 18 hours with MSU crystals (1 mg/mL) with or without α-MSH (10^-6 ^M) or (CKPV)_2 _(10^-6 ^M). Cytokine and caspase-1 protein concentrations were measured in the cell-free supernatants. Bars denote mean ± standard error of the mean. *P < 0.05; **P < 0.02; ***P < 0.01 versus MSU. IL, interleukin; TNF-α, tumor necrosis factor-alpha.

Complete analysis of the molecules responsible for the chemotactic and priming effects in the SMMs was beyond the scope of this study. We focused on IL-1β, TNF-α, and IL-8, the most important cytokines in gouty inflammation. As shown in Figure 4, MSU crystals stimulated production of IL-1β, TNF-α, and IL-8 by Mos. Preincubating SMMs with anti-IL-8 plus anti-IL-1β mAbs significantly reduced, but did not abolish, their chemotactic properties (62% inhibition); as shown in Figure 1, this inhibition was induced mainly by anti-IL-8 mAbs (43% inhibition with anti-IL-8 and 18% inhibition with anti-IL-1β mAb).

**Figure 5:**
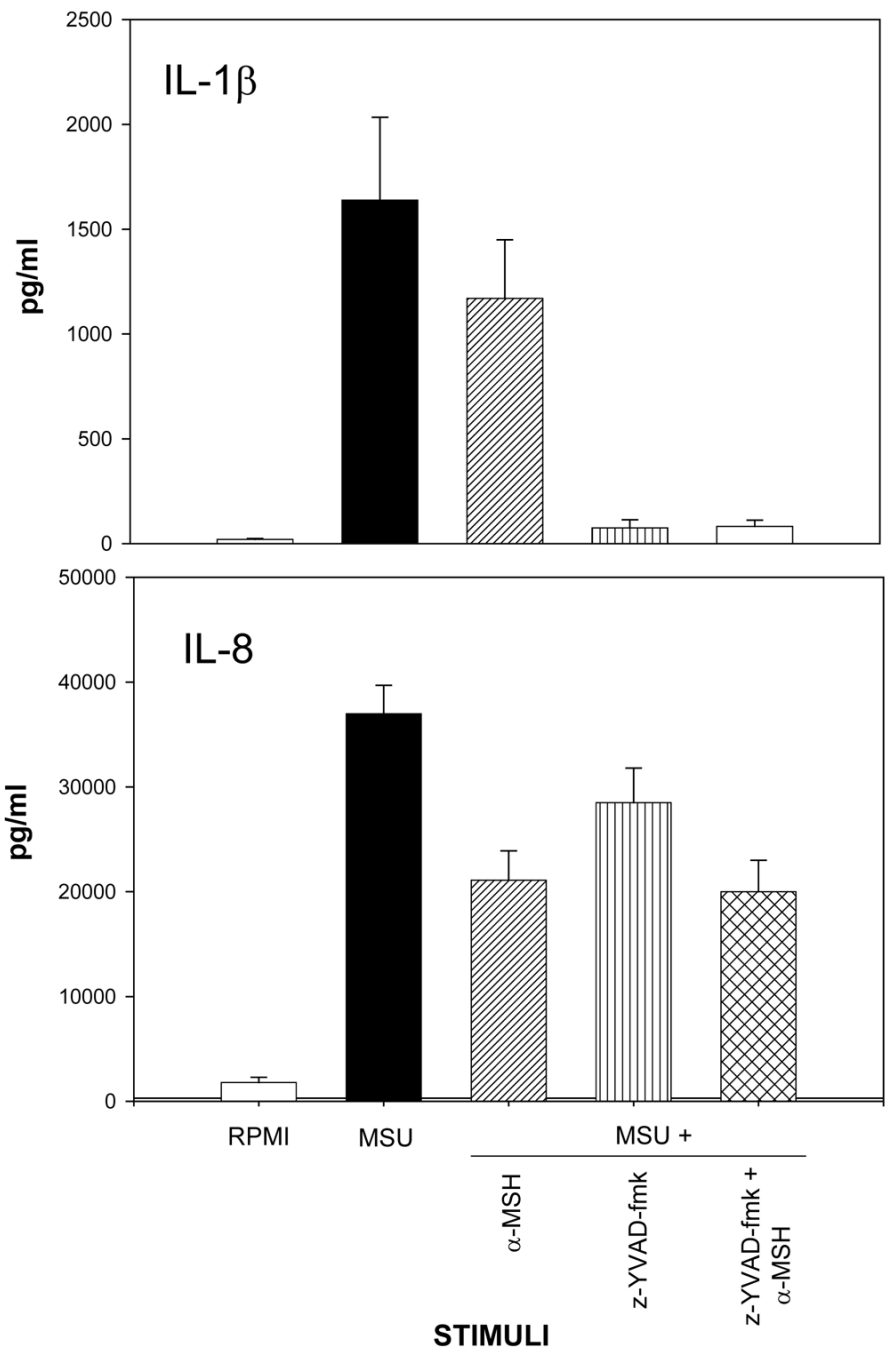
Effect of the caspase-1 inhibitor z-YVAD-fmk on inflammatory mediator production by monosodium urate (MSU)-stimulated human monocytes.  Monocytes were incubated for 18 hours with MSU crystals (1 mg/mL) with or without z-YVAD-fmk (10 mM) or alpha-melanocyte-stimulating hormone (α-MSH) (10^-6 ^M) or the two together. Interleukin (IL)-1β and IL-8 concentrations were measured in the cell-free supernatants. Means ± standard errors of the mean of three separate experiments are presented.

In three separate experiments, IL-1β and IL-8 levels were measured in supernatants from cultured Mos stimulated with MSU in the presence of the caspase-1 inhibitor z-YVAD-fmk. As shown in Figure 5, z-YVAD-fmk completely blocked MSU-induced IL-1β production (95% inhibition) whereas IL-8 secretion was only partially inhibited (23% inhibition).

### Melanocortin peptides reduce the production of neutrophil-activating mediators by monosodium urate-stimulated monocytes

The SMMs obtained in the presence of α-MSH or (CKPV)_2 _showed significant drops in their chemotactic capacity (Figure 1) and priming activity. CL production (Figure 2) and CD11b membrane expression (Figure 3) were significantly lower than in supernatants obtained without the peptides. The capacity of Mos to produce proinflammatory cytokines in response to MSU was reduced by α-MSH and (CKPV)_2_, whereas the secretion of caspase-1, the enzyme responsible for converting pre-IL-1β to the active form of the cytokine, was not affected (Figure 4, bottom right). α-MSH did not further affect the inhibitory activity of z-YVAD-fmk on IL-1β production by MSU-stimulated Mos but did induce further moderate inhibition of IL-8 production (30%) (Figure 5).

### Melanocortin peptides inhibit neutrophil responses to activating mediators produced by monosodium urate-stimulated monocytes

**Figure 6:**
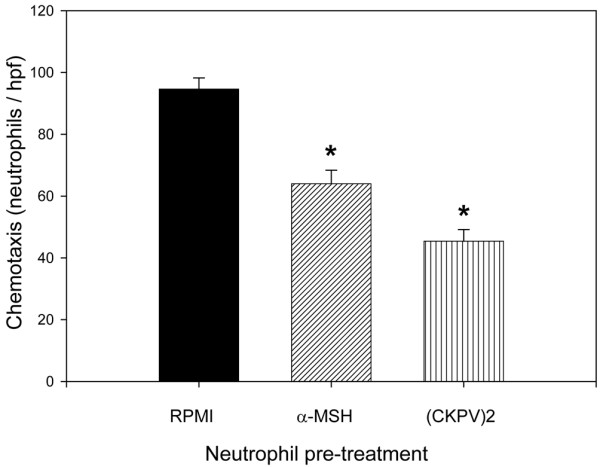
Effect of alpha-melanocyte-stimulating hormone (α-MSH) and (CKPV)_2 _on neutrophil chemotaxis induced by supernatants from monosodium urate-stimulated monocytes.  Neutrophils were pretreated with or without the peptides (10^-6 ^M) for 18 hours before functional assays. Results are shown as number of cells per high-power field (hpf). Means ± standard errors of the mean of five separate experiments are presented. *P < 0.01 versus RPMI.

**Figure 7:**
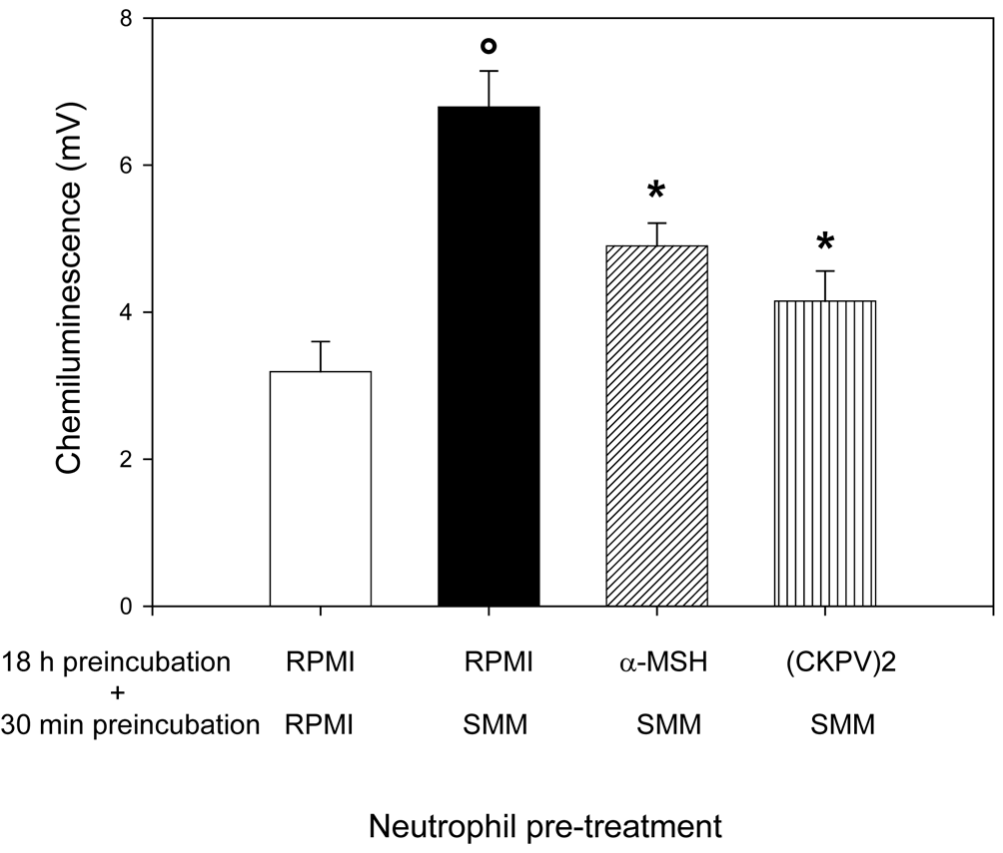
Effects of alpha-melanocyte-stimulating hormone (α-MSH) and (CKPV)_2 _on the priming activity of supernatants from monosodium urate-stimulated monocytes (SMMs) on chemiluminescence (CL) production by neutrophils.  Neutrophils were pretreated with or without the peptides (10^-6 ^M) for 18 hours and then were incubated with RPMI or SMMs for 30 minutes, and CL production in response to monosodium urate (3 mg/mL) was measured. Results are expressed as peak CL values in millivolts. °*P *< 0.01 versus RPMI+RPMI; *P < 0.01 versus RPMI+SMMs.

In parallel experiments, we examined the activity of the melanocortin peptides on neutrophil responses to the SMMs. Neutrophils were preincubated overnight with α-MSH or (CKPV)_2 _before the functional assays. As shown in Figure 6, this pretreatment significantly inhibited the neutrophils' ability to migrate toward the SMMs whereas there was no effect on MSU crystal-induced ROI production (data not shown). However, the overnight pretreatment made the cells less capable of being primed by the SMMs in terms of ROI production (Figure 7).

## Discussion

SMMs exert chemoattractant and priming activity on neutrophils, but SMMs obtained in the presence of the melanocortin peptides had significantly less chemoattractant activity for neutrophils and less ability to prime neutrophils for CD11b membrane expression and the oxidative burst in response to MSU crystals. Stimulation of Mos with MSU crystals induces the production of proinflammatory and chemotactic substances [23-27]. Our interest was focused on IL-1, IL-8, and TNF-α, the cytokines primarily involved in MSU crystal-mediated inflammation. In the presence of the melanocortin peptides, MSU crystal-stimulated Mos produced lower concentrations of these cytokines. Inhibition of these key mediators is probably at least partly responsible for the lower chemotactic and activating properties of the supernatants. Indeed, anti-IL-1β and anti-IL-8 mAbs showed similar inhibitory action.

The present experiments do not clarify the mechanism of the melanocortin peptides' inhibitory effect on Mos' ability to produce chemotactic and activating substances in response to MSU. Recent observations indicate that MSU crystals may act as danger signals that can activate caspase-1 in an NALP-3 inflammosome-dependent manner with production and release of active IL-1β [13]. In this model, the second phase of the inflammatory response is driven by IL-1β/IL-1R signaling and MyD88-dependent NF-κB activation [14]. Consequently, inflammatory mediators that elicit neutrophil recruitment characteristic of acute gouty inflammation are produced.

In line with previous research [13], the present data indicate the production and secretion of caspase-1 by MSU crystal-stimulated Mos. Although melanocortin markedly reduced the release of proinflammatory cytokines, the release of caspase-1 was unaffected. This suggests that melanocortin peptides do not influence the inflammosome-dependent phase of MSU crystal stimulation. This is borne out by the observation that α-MSH still had inhibitory activity on IL-8 secretion by MSU-stimulated Mos in the presence of the caspase-1 inhibitor z-YVAD-fmk.

Two molecular mechanisms by which melanocortin peptides produce their anti-inflammatory effects might be relevant in this experimental model of MSU-induced phagocyte stimulation. First, the peptides prevent activation of NF-κB by a variety of inflammatory stimuli (reviewed in [4]). Therefore, they may inhibit MSU crystal-induced secretion of chemoattractants and activating substances by inhibiting the second phase of the inflammatory response mediated by NF-κB activation. Second, α-MSH and the tripeptide KPV potently and selectively reduce membrane binding of IL-1β to T-cell clones [28,29]. This mechanism could be important in our experimental model as the IL-1β/IL-1R interaction is vital for MSU crystal-induced inflammation [14].

Preincubation of neutrophils with α-MSH or (CKPV)_2 _reduced their ability to migrate toward MSU crystal-induced supernatants and to be primed by SMMs, in terms of ROI production. This agrees with previous observations [9] that melanocortin peptides inhibit neutrophil chemotaxis toward FMLP and IL-8 and their capacity to generate ROIs in response to phorbol esters, an effect that is probably related to the peptides' ability to increase cAMP generation in human neutrophils.

## Conclusions

The present experiments indicate that α-MSH and (CKPV)_2 _have a dual effect on MSU crystal-induced inflammation: they prevent Mos from producing neutrophil chemoattractants and activating compounds and inhibit neutrophil responses to these inflammatory substances. These findings agree with the observations of Getting and colleagues [15,16], who found that melanocortins had protective action in a rat model of gouty arthritis, and suggest a further mechanism for the protective effect. Our results indicate α-MSH and related peptides as a potential new class of drugs for the treatment of inflammatory arthritis.

## Abbreviations

α-MSH: alpha-melanocyte-stimulating hormone; BSA: bovine serum albumin; CL: chemiluminescence; ELISA: enzyme-linked immunosorbent assay; FBS: fetal bovine serum; FMLP: *N*-formyl-methionyl-leucyl-phenylalanine; IL: interleukin; LPS: lipopolysaccharide; mAb: monoclonal antibody; Mo: monocyte; MSU: monosodium urate; NF-κB: nuclear factor-kappa-B; PBS: phosphate-buffered saline; ROI: reactive oxygen intermediate; SMM: supernatant from monosodium urate-stimulated monocytes; TLR: Toll-like receptor; TNF-α: tumor necrosis factor-alpha.

## Competing interests

The authors declare that they have no competing interests.

## Authors' contributions

FC conceived the study, participated in conducting monocyte and neutrophil functional assays, and drafted the manuscript. AMO conducted all of the experimental assays. ER conducted the immunofluorescence assays. AC participated in study design and helped to write the manuscript. All authors read and approved the final manuscript.
